# Comparative Mitogenomics of the Assassin Bug Genus *Peirates* (Hemiptera: Reduviidae: Peiratinae) Reveal Conserved Mitochondrial Genome Organization of *P. atromaculatus*, *P. fulvescens* and *P. turpis*

**DOI:** 10.1371/journal.pone.0117862

**Published:** 2015-02-17

**Authors:** Guangyu Zhao, Hu Li, Ping Zhao, Wanzhi Cai

**Affiliations:** 1 Department of Entomology, China Agricultural University, Beijing, China; 2 Daxing No.1 Middle School, Beijing, China; 3 Department of Ornamental Horticulture, China Agricultural University, Beijing, China; 4 College of Environment and Life Sciences, Kaili University, Kaili, Guizhou, China; Tel-Aviv University, ISRAEL

## Abstract

In this study, we sequenced four new mitochondrial genomes and presented comparative mitogenomic analyses of five species in the genus *Peirates* (Hemiptera: Reduviidae). Mitochondrial genomes of these five assassin bugs had a typical set of 37 genes and retained the ancestral gene arrangement of insects. The A+T content, AT- and GC-skews were similar to the common base composition biases of insect mtDNA. Genomic size ranges from 15,702 bp to 16,314 bp and most of the size variation was due to length and copy number of the repeat unit in the putative control region. All of the control region sequences included large tandem repeats present in two or more copies. Our result revealed similarity in mitochondrial genomes of *P. atromaculatus*, *P. fulvescens* and *P. turpis*, as well as the highly conserved genomic-level characteristics of these three species, e.g., the same start and stop codons of protein-coding genes, conserved secondary structure of tRNAs, identical location and length of non-coding and overlapping regions, and conservation of structural elements and tandem repeat unit in control region. Phylogenetic analyses also supported a close relationship between *P. atromaculatus*, *P. fulvescens* and *P. turpis*, which might be recently diverged species. The present study indicates that mitochondrial genome has important implications on phylogenetics, population genetics and speciation in the genus *Peirates*.

## Introduction

Peiratinae, a subfamily of the assassin bugs (Reduviidae), consists of over 350 described species in 30 valid genera [1]. These assassin bugs are primarily ground-dwelling and are usually attracted to lights [2]. Some of them are common predators in the crop fields and recognized as natural enemies of pest insects. *Peirates* Serville sensu stricto is one of the largest genera of Peiratinae with about 40 valid species worldwide, mainly distributed in the Oriental, Palearctic and Ethiopian regions [3]. Seven species of the genus have been recorded in China [4]. Taxonomic, phylogenetic and biogeographical studies of this genus have been conducted [5–7]. Cladistic analysis based on 51 morphological characters showed three distinct groups in this genus: the *P*. *quadrinotatus* group, the *P*. *singularis* group and the *P*. *lepturoides* group [7]. However, the application of molecular data to the study of this genus is nearly negligible to date, with only one complete mitochondrial (mt) genome sequence and 13 partial DNA sequences of two mt (*16S* rDNA and *COI*) and four nuclear (*18S*, 2*8S* rDNA, *Wingless* and *Deformed*) genes from two species available at GenBank (October 17, 2014).

The mt genome of insect typically contains 13 protein-coding genes (PCGs), 22 transfer RNAs (tRNAs), two ribosomal RNAs (rRNAs) (the large and small ribosomal subunits), and a putative control region [8]. The whole mt genome sequences not only are more informative than individual genes, but also provide a suite of genome level characters [9], such as the gene rearrangement [10–13], strand asymmetry in nucleotide composition [14] and evolutionary patterns of the control region [15,16]. Mt genomes have been applied to a wide array of studies, including molecular systematics at both deep and shallow taxonomic scales [17–20], population genetics [21,22], diagnostics [23] and molecular evolutionary studies [24–26].

The mt genome of *P*. *arcuatus* has been reported previously [27]. Here, we present complete mt genomes of other four *Peirates* species, *P*. *lepturoides*, *P*. *atromaculatus*, *P*. *fulvescens* and *P*. *turpis*, and provide comparative mitogenomic analyses of five species from this genus, e.g., gene order, nucleotide composition, codon usage, tRNA secondary structure, gene overlaps and non-coding regions, to explore the sequence variability and evolutionary traits of the *Peirates* mt genomes.

## Materials and Methods

### Ethics statement

No specific permits were required for the insects collected for this study. The insect specimens were collected from farmland and orchard. The field collections did not involve endangered or protected species. The species sequenced in the family Reduviidae are common insects and are not included in the "List of Protected Animals in China".

### Samples and DNA extraction

Four adult assassin bugs used in this study were collected from the field in China, and the collection information was provided in S1 Table. Specimens were initially preserved in 100% ethanol in the field, and then stored at -20°C. Specimens were identified by the use of taxonomic keys [5, 28] and careful comparisons using morphological characters, especially the color pattern of pronotum and hemelytron (S2 Table; S1 Fig.). For each species, genomic DNA was extracted from adult muscle tissues of the thorax using the DNeasy DNA Extraction kit (Qiagen). Samples and voucher specimens were deposited in the Entomological Museum of China Agricultural University with unique numbers (S1 Table).

### PCR amplification and sequencing

For each species, whole mt genome was amplified by PCR in overlapping fragments with universal insect mt primers [29] (S3 Table), and species-specific primers were designed based on the sequenced fragments to bridge gaps. PCR and sequencing reactions were conducted following [12,16,30]. The sequence data have been deposited in GenBank (Table 1).

**Table 1:** Structural feature of *Peirates* mitochondrial genomes used in this study.

Size (bp)										
Species	GenBank ^a^	AT%	AT-skew	GC-skew	Genome	PCGs	tRNAs	srRNA	lrRNA	CR ^b^
*P*. *fulvescens* *	KF913537	71.9	0.149	-0.181	15,702	11,143	1,447	784	1,253	1,044
*P*. *atromaculatus* *	KF913539	71.0	0.151	-0.195	16,151	11,143	1,451	784	1,254	1,488
*P*. *turpis* *	KF913540	72.0	0.147	-0.183	15,703	11,143	1,448	784	1,253	1,044
P. arcuatus	KF752445	71.3	0.128	-0.176	16,176	11,114	1,456	781	1,254	1,552
*P*. *lepturoides* *	KF913541	72.4	0.13	-0.158	15,932	11,146	1,461	781	1,261	1,263

* mitochondrial genome sequenced in this study

^a^ GenBank accession number

^b^ control region.

### Genome assembly and annotation

Contigs were assembled from forward and reverse reads using Sequencher (Gene Codes Corporation, Ann Arbor, MI, USA). Protein-coding genes and rRNA genes were identified by BLAST searches in GenBank and subsequently confirmed by alignment with homologous genes from other true bugs. tRNA genes were identified by tRNAscan-SE Search Server v.1.21 [31]. *trnR* and *trnS1*, which could not be identified by tRNAscan-SE, were determined by sequence similarity comparison with tRNA genes of other insects.

### Sequence alignment and genomic analyses

Each PCG was aligned individually based on codon-based multiple alignments by using the MAFFT algorithm within the TranslatorX [32] online platform. The sequences of tRNAs, rRNAs and non-coding regions were aligned respectively using ClustalW in MEGA 5.0 [33]. The base composition, codon usage and genetic distances of PCGs were analyzed with MEGA 5.0. Sequence alignments of barcoding region, single PCG and concatenated 13 PCGs were provided in S1 Dataset.

### Phylogenetic analyses

Complete mt genomes of five species in the genus *Peirates* and a outgroup species *Sirthenea flavipes* (GenBank: NC_020143) from the same subfamily were used in phylogenetic analyses. We inferred phylogenies using sequences of all 37 mt genes (13 PCGs, two rRNAs and 22 tRNAs). Alignments of individual genes were concatenated using SequenceMatrix v1.7.8 [34]. The concatenated matrix with 14,692 nucleotides was analyzed with maximum likelihood (ML), Bayesian inference (BI) and neighbor-joining (NJ) methods.

The optimal partition strategy and model of each partition was selected by PartitionFinder v1.1.1 [35]. We created an input configuration file that contained pre-define 37 gene partitions. The ‘‘greedy’’ algorithm with branch lengths estimated as ‘‘unlinked’’ and Bayesian information criterion (BIC) was used to search for the best-fit scheme.

We performed ML and BI analyses using the best-fit partitioning schemes recommended by PartitionFinder (S4 Table). ML analyses were conducted using RAxML 8.0.0 [36] with GTRGAMMA model. Node support was calculated by acquiring bootstrap values from heuristic searches of 1000 resampled datasets, using the rapid bootstrap feature (random seed value 12345) [37]. Bayesian analyses were carried out using MrBayes 3.2.2 [38]. Two simultaneous runs of 5 million generations were conducted for the dataset and trees were sampled every 1000 generations, with the first 25% discarded as burn-in. Two runs have satisfactorily converged with standard deviation of split frequency lower than 0.0001. All RAxML and MrBayes analyses were conducted in the CIPRES Science Gateway v3.3 [39]. The neighbor-joining tree was constructed using MEGA 5.0 [33] with 1000 bootstrap replicates. NEXUS and PHYLIP file for BI and ML analyses were provided in S2 and S3 Datasets.

## Results and Discussion

### General features of Peirates mt genomes

**Fig 1:**
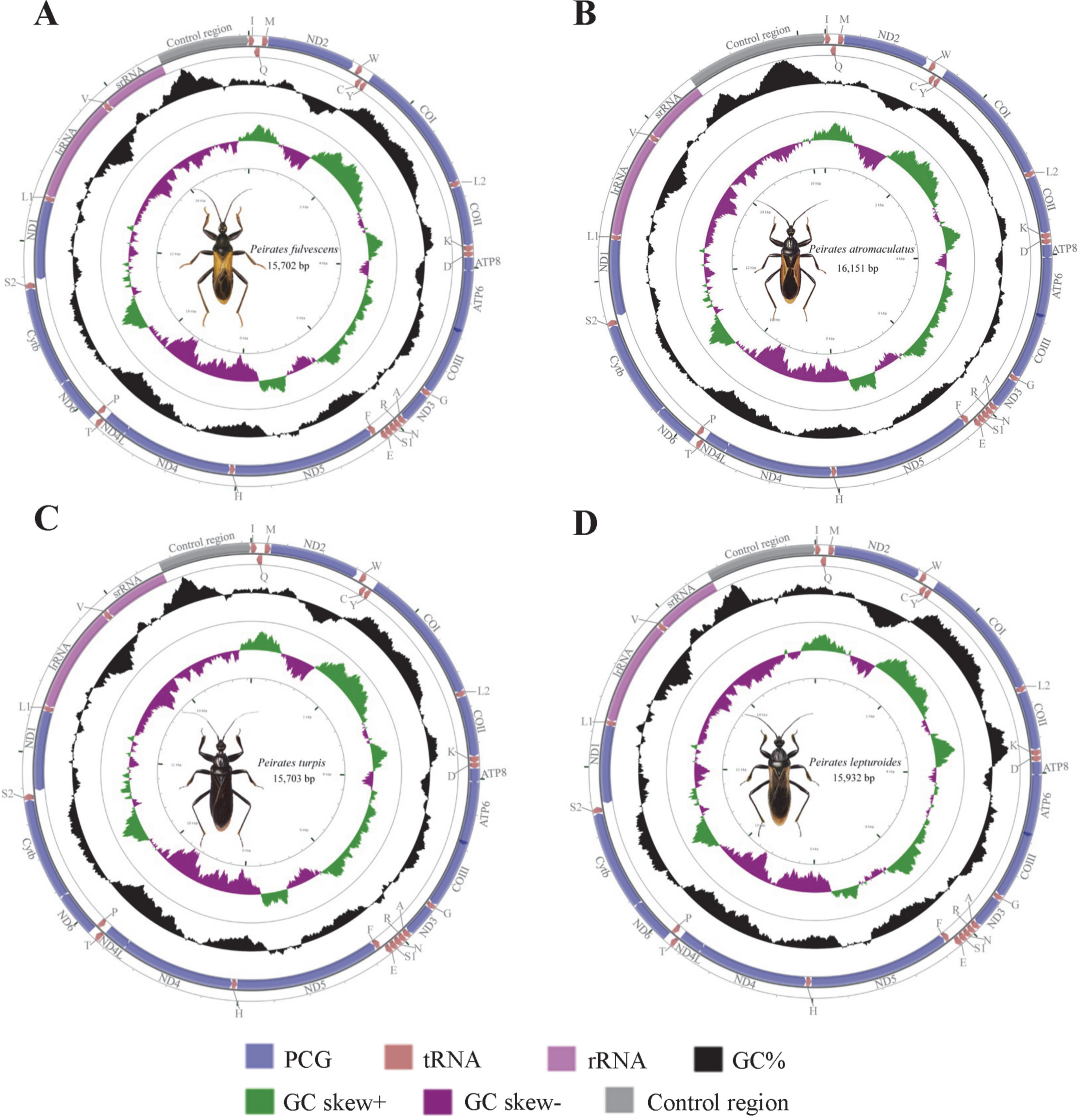
Mitochondrial maps of four sequenced assassin bugs. Direction of gene transcription is indicated by arrows. PCGs are shown as blue arrows, rRNAs as purple arrows, tRNAs as red arrows and large NC regions (>100 bp) as grey arrows. tRNAs are labeled according to single-letter IUPAC-IUB abbreviations (L1: UUR; L2:CUN; S1:AGN; S2:UCN). The GC content is plotted using a black sliding window, as the deviation from the average GC content of the entire sequence. GC-skew is plotted as the deviation from the average GC-skew of the entire sequence. Ticks in the inner cycle indicate the sequence length.

In this study, mt genomes of four species were newly sequenced, and five complete mt genomes representing five species in the genus *Peirates* were compared (Fig. 1, Table 1). For convenience, we assigned each mt genome an abbreviation (PF for *P*. *fulvescens*, PAY for *P*. *atromaculatus*, PT for *P*. *turpis*, PL for *P*. *lepturoides* and PA for *P*. *arcuatus*). These mt genomes ranged in size from 15,702 bp for PF to 16,176 bp for PA and most of the size variation was due to differences in the putative control region. All of the genomes examined here had a typical set of 37 genes and retained the so-called ancestral mt genome arrangement of insects (Fig. 1). Generally, the A+T content, AT- and GC-skews exhibited similar behaviors and were similar to the common base composition biases of insect mtDNA [14]. Some general characteristics of the genomes were given in Table 1.

### Protein-coding genes

All PCGs of the analyzed mt genomes initiated with ATN start codons and mostly terminated with TAA or TAG stop codons, except for several genes ended with a single T residue adjacent to a downstream tRNA gene, e.g., *COII*, *COIII*, *ND3* and *ND5* (S5 Table). The start and stop codons were identical in three assassin bugs (PF, PAY and PT), but showed differences between them and other two species in some PCGs (S5 Table). The non-stop codon usage also showed the same pattern and the total numbers of non-stop codons were identical in these three species (3,704 codons) but exhibited different numbers in other two species (S2 Fig.)

For a better understanding of the sequence divergence of PCGs in *Peirates*, we calculated the genetic distances within five assassin bugs based on barcoding region of *COI*, single PCG and concatenated 13 PCGs. The final alignment length for barcoding region was 665 bp. Kimura-2 parameter pairwise genetic distance revealed low variation among PF, PAY and PT (averaged 3.1%, range 1.5–4.1%) and high sequence divergence between these three assassin bugs and two other species (averaged 13.8%, range 11.9–16.1%) (S6 Table). Concatenated 13 PCGs and most of the single PCGs exhibited similar genetic distances with barcoding region, while *ATP8*, *ND1*, *ND2 and ND3* showed relatively higher distances than barcoding region (S6 Table). In general, the genetic distances based on barcoding region, single PCG and concatenated 13 PCGs showed consistent result that the sequence divergence of PF, PAY and PT was very low.

### Transfer and ribosomal RNAs

**Fig 2:**
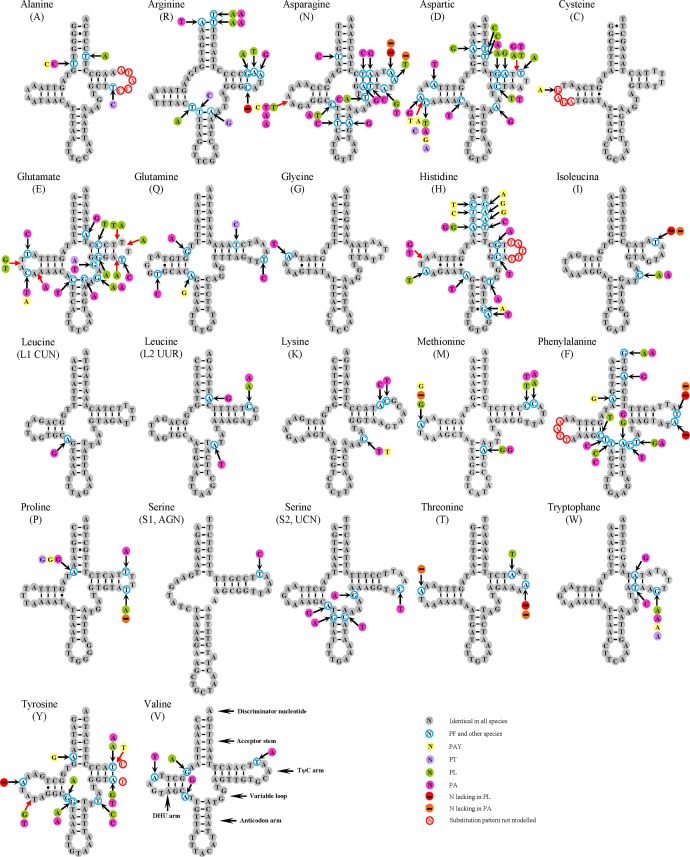
Secondary structure of tRNA families in *Peirates* mitochondrial genomes. The nucleotide substitution pattern for each tRNA family is modeled using as reference the structure determined for PF. Red arrows correspond to insertions. The tRNAs are labeled with the abbreviations of their corresponding amino acids. Inferred Watson-Crick bonds are illustrated by lines, whereas GU bonds are illustrated by dots.

All of the 22 typical animal tRNA genes were found in *Peirates* mt genomes, ranging from 63 to 70 bp in length (Fig. 2). Most of the tRNAs could be folded into the classic cloverleaf secondary structure except for *trnS1*, in which its dihydrouridine (DHU) stem simply formed a loop. The loss of the DHU stem in *trnS1* is a common feature in assassin bugs [30,40,41] as well as other insects [26,42,43].

According to the secondary structures and sequence alignment, the most conserved tRNAs in *Peirates* mt genomes were *trnG*, *trnL1* and *trnS1* with one nucleotide substitution in each gene (Fig. 2). In the remaining tRNAs, nucleotide substitutions were mainly restricted to TΨC and DHU loops, with obvious insertion-deletion polymorphisms. In *trnA*, *trnC*, *trnH*, *trnF* and *trnY*, the substitution pattern of the TΨC or DHU loop was difficult to model due to a high level of variation among orthologous sequences. Interestingly, tRNAs of three assassin bugs (PF, PAY and PT) showed high level of sequence and structural identity, with eight identical tRNAs (*trnG*, *trnI*, *trnL1*, *trnL2*, *trnS1*, *trnS2*, *trnT* and *trnV*) (S3 and S4 Figs.). The fourteen remaining tRNAs only had four insertion-deletion positions in the DHU arm in total, and the nucleotide substitutions of individual gene were mostly restricted to 1–3 nucleotide sites, with the exception of *trnH* with 6 sites (S3 Fig.).

Genes for the small and large subunit ribosomal RNAs (*srRNA* and *lrRNA*) were adjacent on the same strand, interleaved by a single *trnV*. The size differences in both ribosomal subunits were not distinct among different species, especially in PF, PAY and PT (Table 1). The multiple alignments of two rRNAs displayed consistent results with tRNAs that sequences of three species (PF, PAY and PT) showed extremely higher identity (S5 Fig.).

### Gene overlaps and non-coding regions

All five mt genomes had gene overlaps and the size ranged from 1 to 44 bp (S7 Table). The number of gene overlaps in PA (12 overlaps) was identical with that in three assassin bugs (PF, PAY and PT), and the fewest number was found in PL (8 overlaps). Except for the common overlap between *ATP8-ATP6* in Heteroptera [16], all five mt genomes shared three pairs of gene overlaps: *trnW-trnC* (8 bp), *COI-trnL2* (5 bp) and *ND6-CytB* (1 bp), which may be the common features in the genus *Peirates*. All gene overlaps in three assassin bugs (PF, PAY and PT) had the same locations and sequences of each overlap were almost identical, except for the overlap between *ATP6-COIII* in PAY with a nucleotide substitution.

Outside control region, there were 9 non-coding regions in the mt genomes of PF, PAY and PT, 10 in PL and 11 in PA (S8 Table). Four non-coding regions, *trnQ-trnM* (22 bp), *trnY-COI* (1 bp), *trnA-trnR* (3 bp) and *trnS2*-*ND1* (22 bp), were observed in all mt genomes. It is worth noting that the pattern of non-coding regions was similar to that of gene overlaps in three species (PF, PAY and PT), sharing the same number and distribution of genomic spacers, the same size and almost identical sequences of individual non-coding region.

### Control region

**Fig 3:**
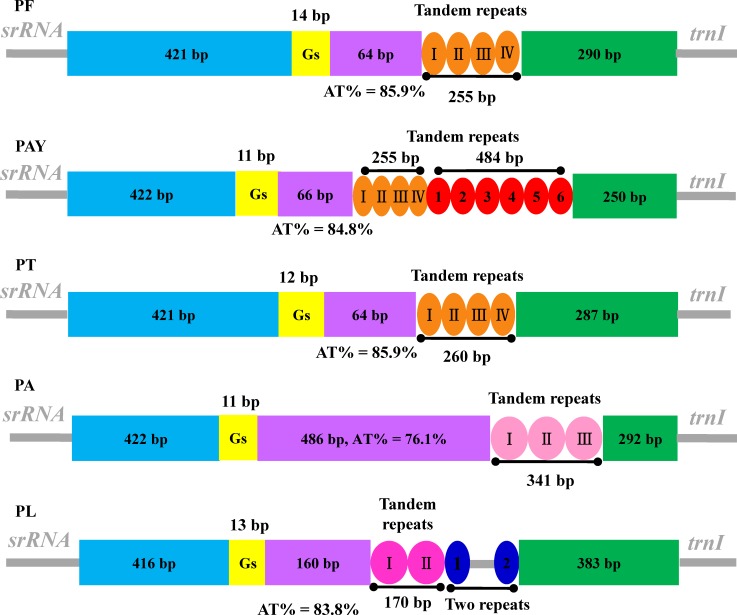
Organization of the control region in *Peirates* mitochondrial genomes. The colored panes indicate the structural elements in the CR, leading sequences are shown as blue panes, strings of Gs as yellow panes, A+T-rich sequences as purple panes and the remainders of control regions as green panes.

The putative control region, which included the presumed origin of replication and promoters for transcription initiation [15,44], located between *srRNA* and *trnI*. The size of this region was relatively variable, ranging from 1,044 bp in PF to 1,552 bp in PA. The following structural elements were summarized in the control region of *Peirates* mt genomes: (1) a leading sequence adjacent to *srRNA*, (2) a string of Gs, (3) an A+T-rich sequence block, (4) a tandem repeated sequence block consisting of repeat units, and (5) the remainder of the control region (Fig. 3).

Unlike coding regions, control regions from different insect species always exhibit a very high level of divergence [15,45]. Comparison of the nucleotide sequences of structural elements (Gs and tandem repeats were not included) in control region showed the high level of sequence conservation in three assassin bugs (PF, PAY and PT), with nucleotide identity ranged from 91.13% for the remainder of control region to 98.5% for leading sequence; and extremely low sequence identity in all five species, ranging from 15.47% for AT-rich sequence to 70.37% for leading sequence (S9 Table).

Variation of size and copy number of the repeat unit is responsible to a large degree for the size variation of the control region [15,45,46]. All of the control region sequences examined here included large tandem repeats present in two or more copies (Fig. 3). The complete sequences of PF, PAY and PT shared a nearly identical tandem unit, a 71 bp sequence tandemly repeated three times, with a partial forth. In addition, another type of tandem unit (89 bp) with five copies and a partial sixth was found in PAY. Conversely, the other two species exhibited totally different tandem units, two 118 bp tandem repeat units and a partial third were found in PA, and PL had two 85 bp tandem repeat units and two 39 bp repeats. Collectively, mt control region of the genus *Peirates* had several distinct structural and evolutionary characteristics, including variable size, conserved structural elements, and abundant tandem repetitions. These properties of control region were also discovered in other assassin bugs [30,40,41,47,48] as well as other true bugs [12,16,45,49–51], and made this region an ideal molecular marker for evolutionary and population genetic studies.

### Phylogenetic analyses

**Fig 4:**
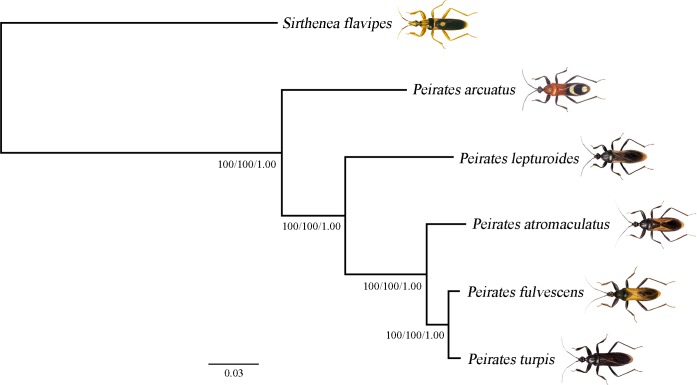
Phylogenetic relationships among *Peirates* assassin bugs inferred from 37 mitochondrial genes. Phylogram from the Bayesian analysis of partitioned 37 mitochondrial genes is shown. Numbers close to the branching points are bootstrap support values from the ML analysis, bootstrap support values from NJ analysis and posterior probabilities from the Bayesian analysis.

The topologies and node support values of three phylogenetic trees (BI/ML/NJ) inferred from the nucleotide sequences of all 37 mt genes were identical (Fig. 4). In all of the phylogenetic trees constructed with different methods: 1) *P*. *lepturoides*, *P*. *atromaculatus*, *P*. *fulvescens* and *P*. *turpis* were monophyletic; and 2) *P*. *atromaculatus* was the sister-group to *P*. *fulvescens* and *P*. *turpis*. Previous cladistic analysis based on morphological characters showed three distinct groups in *Peirates*: *P*. *quadrinotatus* group, *P*. *singularis* group and *P*. *lepturoides* group [7]. *P*. *lepturoides* group is the largest species group (more than 20 species), including *P*. *lepturoides*, *P*. *atromaculatus* and *P*. *turpis*. Our results suggest that *P*. *fulvescens* may belong to the *P*. *lepturoides* species group. However, more sequences are needed to confirm this result. Although the phylogenetic analysis based on the current taxon was limited to inferring the phylogeny of *Peirates*, it still had important implications for the usefulness of mt genome sequence in the phylogenetic studies of this genus. Furthermore, next-generation sequencing (NGS) technology makes it possible to efficiently and cost-effectively obtain entire mt genome from large number of samples for resolving relationships at different taxonomic levels and population structure of species [17, 52–55].

In conclusion, with four newly sequenced mt genomes from the genus *Peirates*, we presented the first comparative mitogenomic analysis of these assassin bugs. Our results showed that gene content, gene order, base composition and tRNA secondary structures were conserved among *Peirates* mt genomes. Control region possessed several distinct characteristics, including: variable size, abundant tandem repetitions and conserved structural elements, and was useful in evolutionary and population genetic studies of this genus. Phylogenetic and comparative mitogenomic analyses revealed the close relationship of *P*. *atromaculatus*, *P*. *fulvescens* and *P*. *turpis*, as well as the highly conserved mitogenomic organization, e.g., 1) the overall sequence similarity and low genetic distances, 2) same start and stop codons of PCGs, 3) conserved secondary structure of tRNAs, 4) identical location and length of non-coding and overlapping regions, and 5) conservation of structural elements and tandem repeat unit in control region. All these conserved characters indicated that these three close related assassin bugs might be the recently diverged species. In summary, the present study showed the usefulness of mt genome in evolutionary and phylogenetic studies of the genus *Peirates*. More taxon sampling in the future study should help to better understanding the phylogeny and evolution of these assassin bugs.

## Supporting Information

S1 Fig The different color patterns of the hemelytron in five assassin bugs. A, *P*. *fulvescens* (PF); B, *P*. *atromaculatus* (PAY); C, *P*. *turpis* (PT); D, *P*. *lepturoides* (PL); E, *P*. *arcuatus* (PA).
(TIF)

S2 Fig Codon usage of protein-coding genes in *Peirates* mitochondrial genomes. Codon families are provided on the x-axis. Numbers to the right refer to the total number of codons.
(TIF)

S3 Fig Alignment of tRNA families (trnA-trnL2) in mitochondrial genomes of three assassin bugs (PF, PAY and PT). The loop regions are highlighted by the black pane.
(TIF)

S4 Fig Alignment of tRNA families (trnL1-trnV) in mitochondrial genomes of three assassin bugs (PF, PAY and PT). The loop regions are highlighted by the black pane.
(TIF)

S5 Fig Nucleotide variation of two rRNAs in *Peirates* mitochondrial genomes. Three assassin bugs indicate *P*. *atromaculatus*, *P*. *fulvescens* and *P*. *turpis* (PAY, PF and PT).
(TIF)

S1 Table Collection information of *Peirates* species newly sequenced in the present study. (DOCX)

S2 Table Key morphological characters used to species identification in this study. (DOCX)

S3 Table Primer sequences used in this study. (DOCX)

S4 Table The best partitioning scheme selected by PartitionFinder for the BI and ML analyses. (DOCX)

S5 Table Start and stop codons of protein-coding genes in *Peirates* mitochondrial genomes. (DOCX)

S6 Table Genetic distances based on barcoding region, single PCG and concatenated 13 PCGs. (DOCX)

S7 Table Statistics on gene overlaps in *Peirates* mitochondrial genomes. (DOCX)

S8 Table Statistics on non-coding regions in *Peirates* mitochondrial genomes. (DOCX)

S9 Table Sequence identities of structural elements in control region. (DOCX)

S1 Dataset Sequence alignments of barcoding region, single PCG and concatenated 13 PCGs. (TXT)

S2 Dataset NEXUS file for BI analysis. (NEX)

S3 Dataset PHYLIP file for ML analysis. (PHY)
