# Supplementary figures and images for: Comparative Mitogenomics of the Assassin Bug Genus Peirates (Hemiptera: Reduviidae: Peiratinae) Reveal Conserved Mitochondrial Genome Organization of P. atromaculatus, P. fulvescens and P. turpis
Source: PLoS One. 2015 Feb 17;10(2):e0117862. doi: 10.1371/journal.pone.0117862 (PMC4331094; doi:10.1371/journal.pone.0117862)

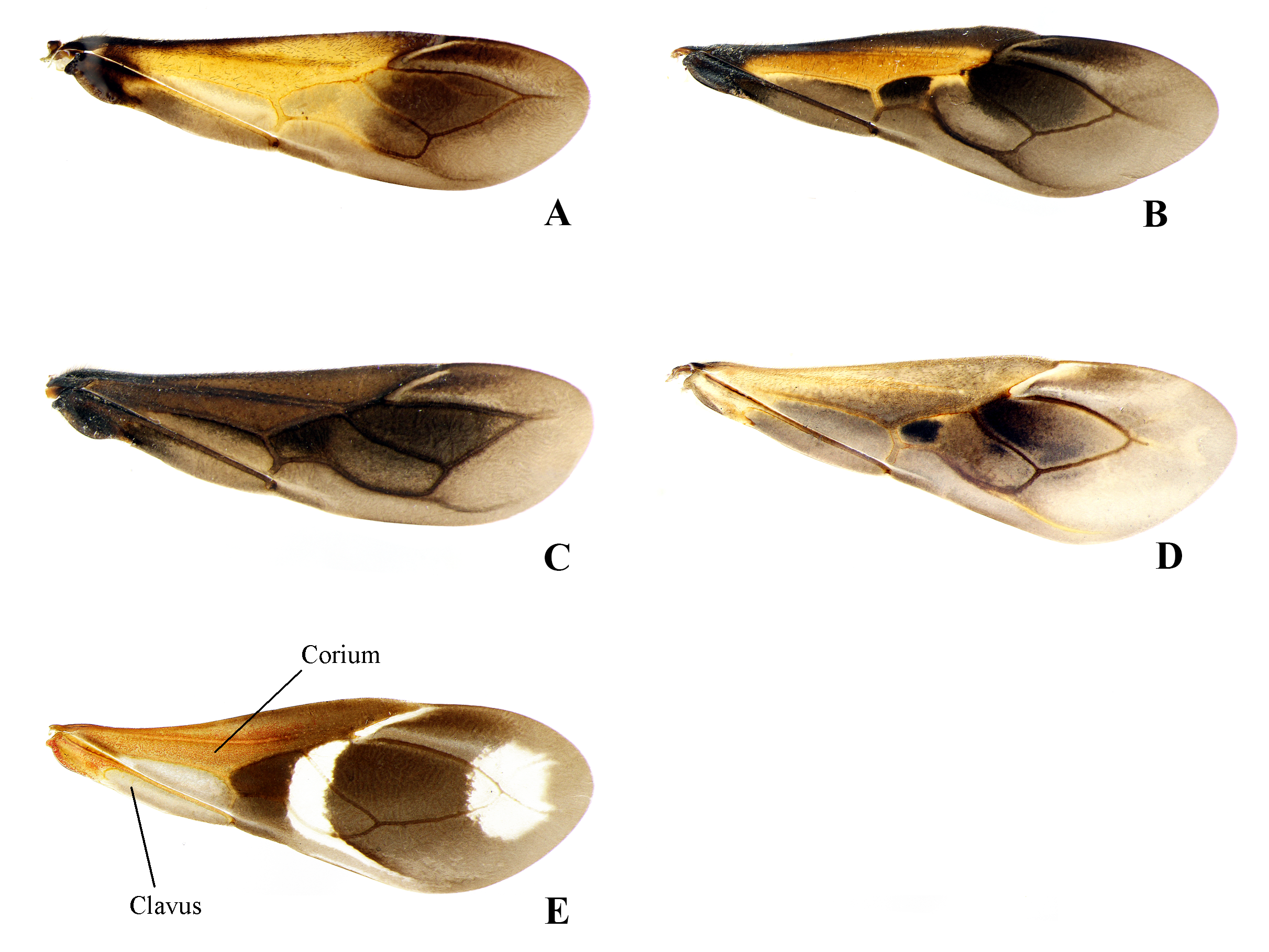

Supplement: S1 Fig — A, P. fulvescens (PF); B, P. atromaculatus (PAY); C, P. turpis (PT); D, P. lepturoides (PL); E, P. arcuatus (PA). (TIF) [file pone.0117862.s001.tif]

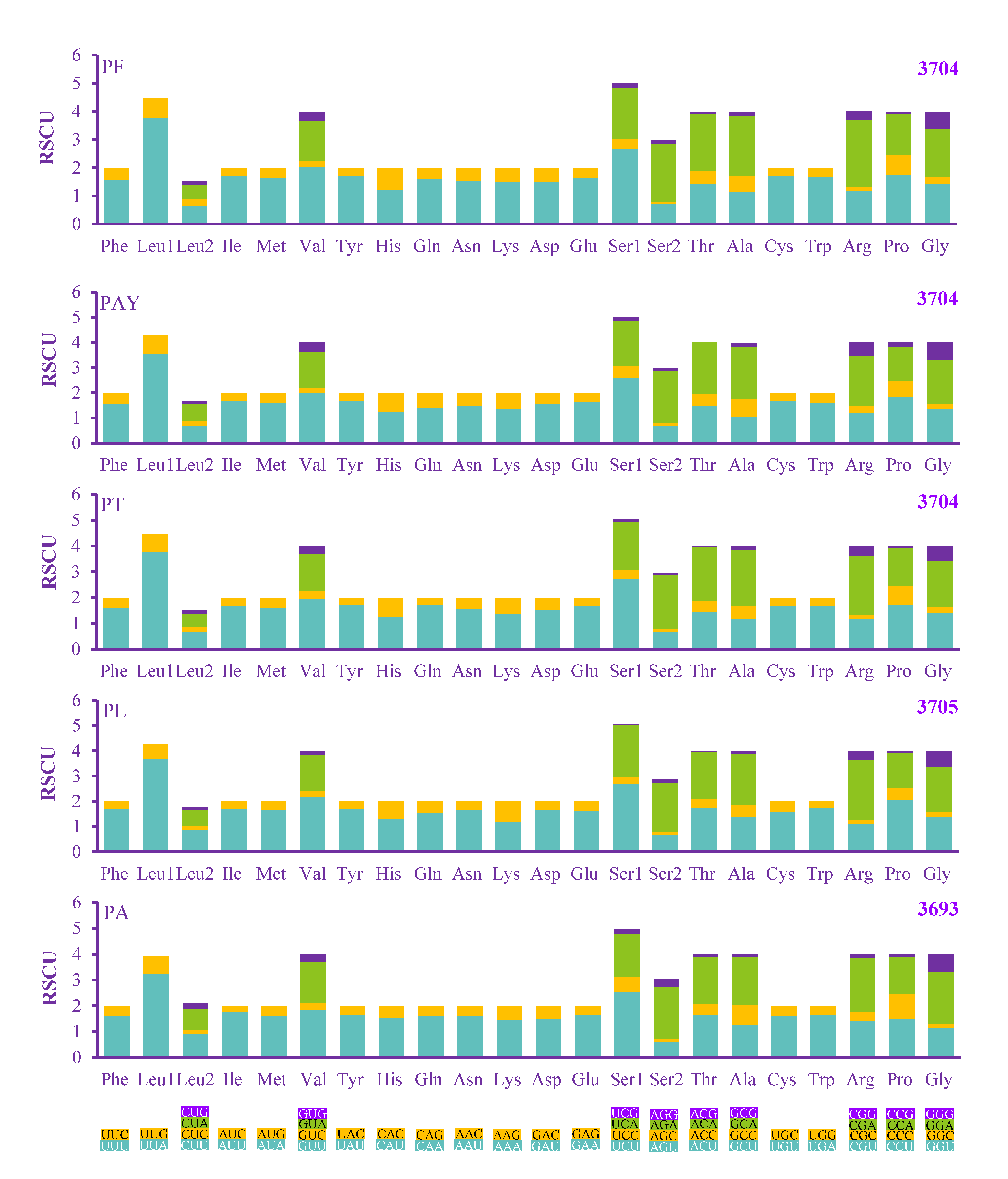

Supplement: S2 Fig — Codon families are provided on the x-axis. Numbers to the right refer to the total number of codons. (TIF) [file pone.0117862.s002.tif]

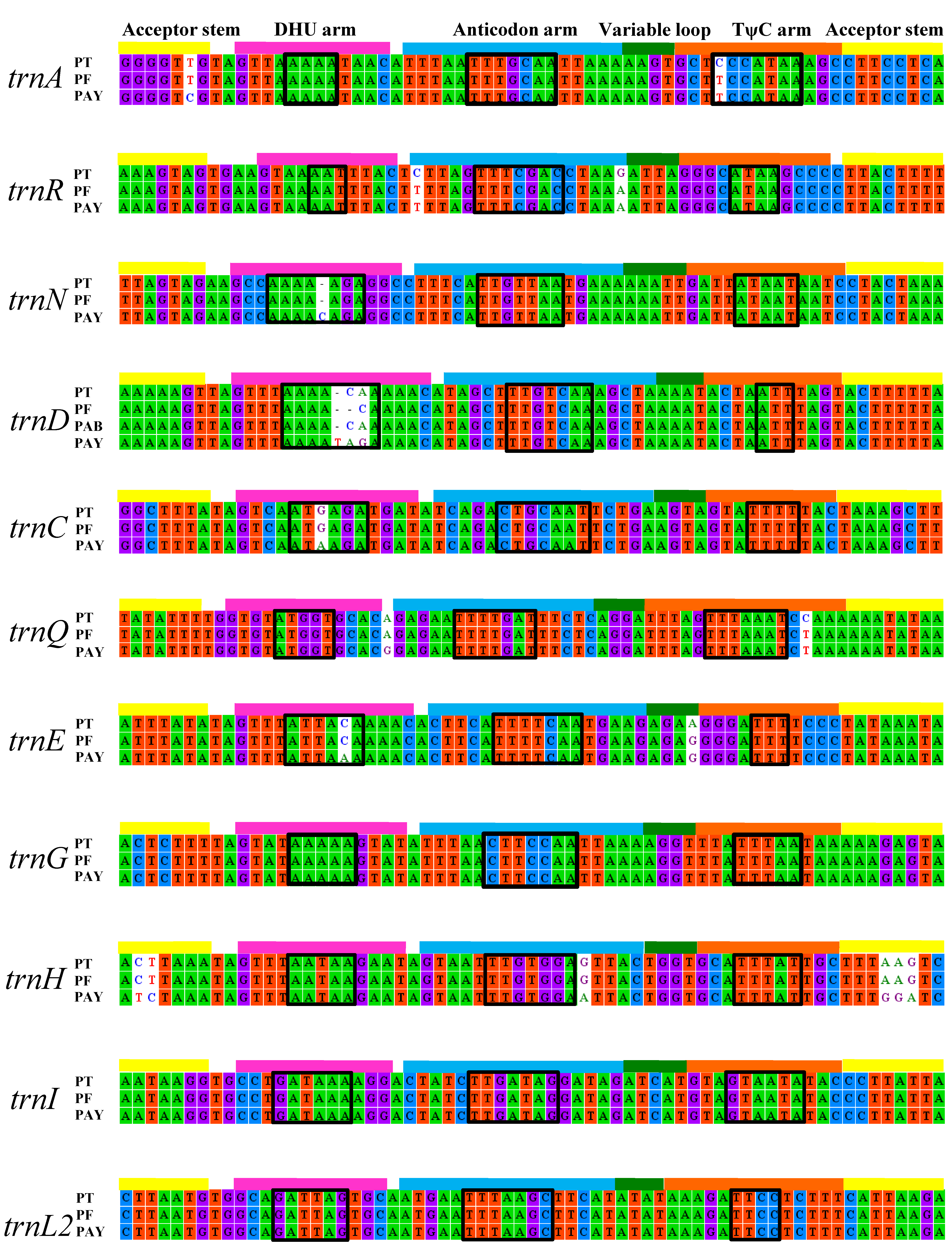

Supplement: S3 Fig — The loop regions are highlighted by the black pane. (TIF) [file pone.0117862.s003.tif]

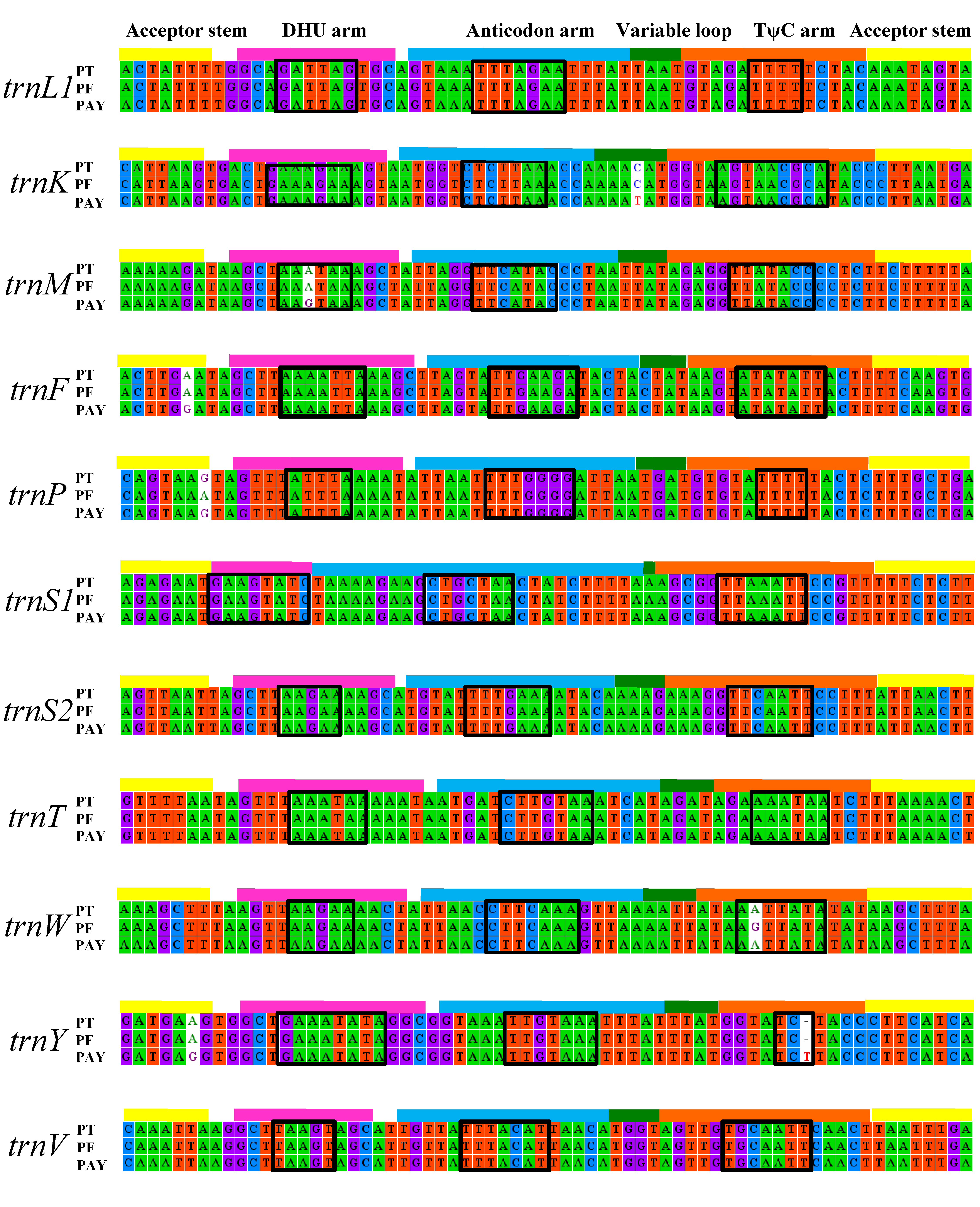

Supplement: S4 Fig — The loop regions are highlighted by the black pane. (TIF) [file pone.0117862.s004.tif]

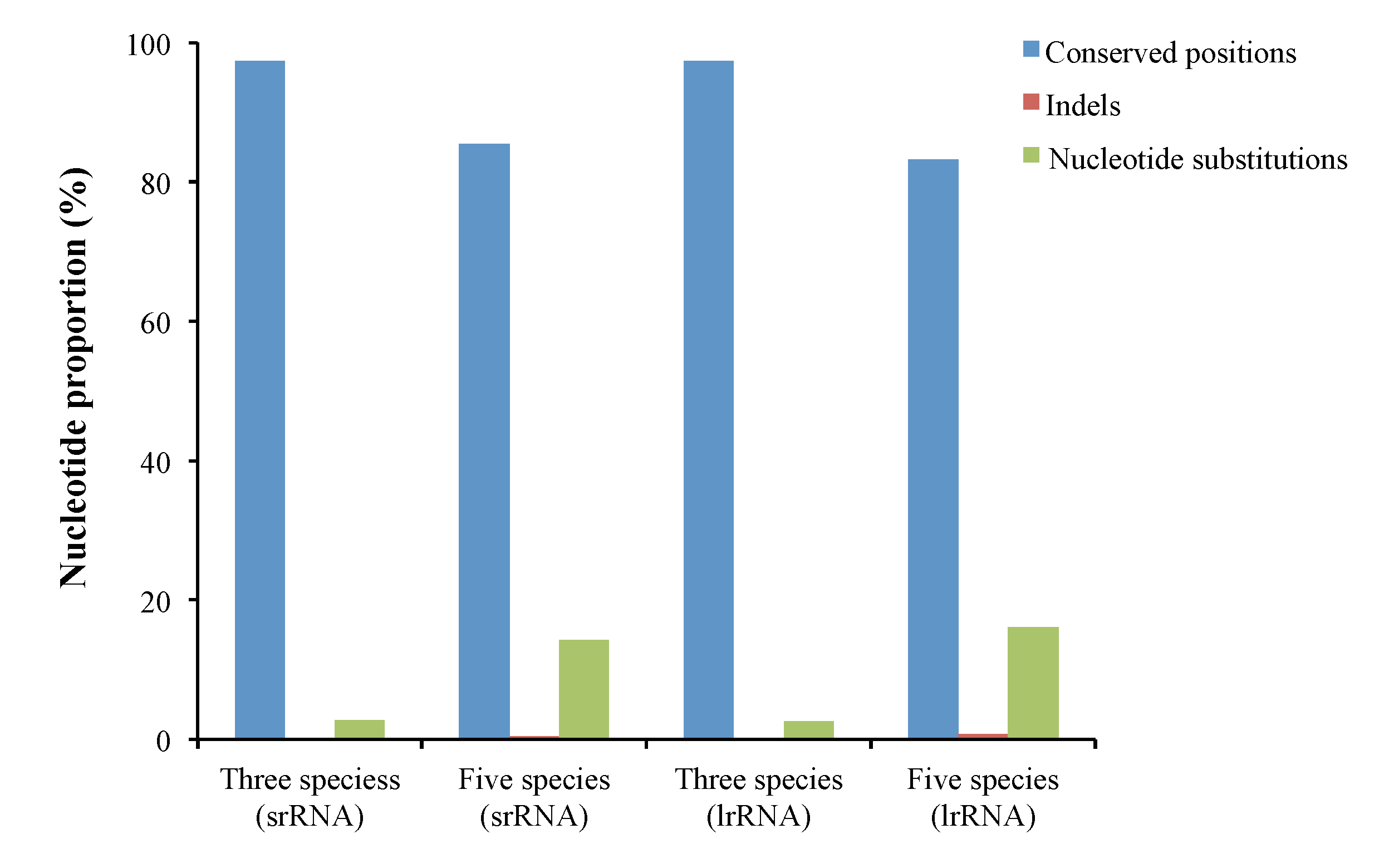

Supplement: S5 Fig — Three assassin bugs indicate P. atromaculatus, P. fulvescens and P. turpis (PAY, PF and PT). (TIF) [file pone.0117862.s005.tif]
