# Supplementary material for: Comparative Mitogenomics of the Assassin Bug Genus Peirates (Hemiptera: Reduviidae: Peiratinae) Reveal Conserved Mitochondrial Genome Organization of P. atromaculatus, P. fulvescens and P. turpis
Source: PLoS One. 2015 Feb 17;10(2):e0117862. doi: 10.1371/journal.pone.0117862 (PMC4331094; doi:10.1371/journal.pone.0117862)
Supplement: S1 Table — (DOCX) [file pone.0117862.s006.docx]

**Table S1 Collection information of *Peirates* species newly sequenced in the present study**

| **Species** | **Abbreviation** | **Locality** | **Time** | **Museum No.** |
| --- | --- | --- | --- | --- |
| *Peirates fulvescens* | PF | Wulingshan, Beijing, China | 6-23-2014 | CAU-MgR1 |
| *Peirates atromaculatus* | PAY | Mengla, Yunnan, China | 5-7-2009 | CAU-MgR3 |
| *Peirates turpis* | PT | Lishan, Shanxi, China | 7-26-2012 | CAU-MgR4 |
| *Peirates lepturoides* | PL | Jinggangshan, Jiangxi, China | 10-8-2012 | CAU-MgR5 |
