# Supplementary material for: Comparative Mitogenomics of the Assassin Bug Genus Peirates (Hemiptera: Reduviidae: Peiratinae) Reveal Conserved Mitochondrial Genome Organization of P. atromaculatus, P. fulvescens and P. turpis
Source: PLoS One. 2015 Feb 17;10(2):e0117862. doi: 10.1371/journal.pone.0117862 (PMC4331094; doi:10.1371/journal.pone.0117862)
Supplement: S2 Table — (DOCX) [file pone.0117862.s007.docx]

**Table S2 Key morphological characters used to species identification in this study.**

|  |  | **Hemelytron ^a^** |  |
| --- | --- | --- | --- |
| **Species** | **Pronotum** | **Corium** | **Clavus** |
| *P. fulvescens* | Black | Almost yellow | Almost yellow |
| *P. atromaculatus* | Black | Area between veins Cu and R + M orange | Black |
| *P. turpis* | Black | Almost black | Black |
| *P. arcuatus* | Brown | Brown and basal half brown with orange tonalities | Brown and basal zone with orange tonalities |
| *P. lepturoides* | Black | Light brown | Upper area light brown |

^a^ Photo of hemelytron for each specimen was shown in Figure S1.
