# Supplementary material for: Comparative Mitogenomics of the Assassin Bug Genus Peirates (Hemiptera: Reduviidae: Peiratinae) Reveal Conserved Mitochondrial Genome Organization of P. atromaculatus, P. fulvescens and P. turpis
Source: PLoS One. 2015 Feb 17;10(2):e0117862. doi: 10.1371/journal.pone.0117862 (PMC4331094; doi:10.1371/journal.pone.0117862)
Supplement: S3 Table — (DOCX) [file pone.0117862.s008.docx]

**Table S3 Primer sequences used in this study**

| **Fragment** | **Primer ID** | **Nucleotide sequence (5’-3’)** |
| --- | --- | --- |
| **1** | SPA-2756 | ACATTTTTTCCTCAACATTT |
|  | SPA-3665 | CCACAAATTTCTGAACACTG |
| **2** | SPA-3399 | TCTATTGGTCATCAATGGTACTG |
|  | SPA-4061 | GAAAATAAATTTGTTATCATTTTCA |
| **3** | SPA-3790 | CATTAAGTGACTGAAAGCAAGTA |
|  | SPA-4552 | ATGACCTGCAATTATATTAGC |
| **4** | SPA-4463 | TTTTATCATCTTATTCCTGTGGG |
|  | SPA-4908 | CGTGTTACATCACGTCATCATTG |
| **5** | SPA-4792 | GTAGATGCAAGCCCTTGACC |
|  | SPA-5731 | ATTGGATCAAATCCACATTC |
| **6** | SPA-5747 | CCATTTGAATGTGGRTTTGATCC |
|  | SPA-6384 | AAAATTAAAAGCATAATATTGAAG |
| **7** | SPA-6172 | AGAGGCAATTTATTGTTAATAA |
|  | SPA-7211 | TTAAGGCTTTATTATTTATATGTGC |
| **8** | SPA-7077 | TTAAATCCTTTGAGTAAAATCC |
|  | SPA-7793 | TTAGGTTGAGATGGTTTAGG |
| **9** | SPA-7572 | AAACGGAAACTGAGCTCTCTTAGT |
|  | SPA-8727 | AAATCTTTAATTGCCTATTCTTC |
| **10** | SPA-8641 | CCAGAAGAACATAGCCCATG |
|  | SPA-9629 | GTTTGTGAAGGTGTGTTGGG |
| **11** | SPA-9648 | TCCCAACACACCTTCACAAAC |
|  | SPA-11010 | TATCAACAGCAAATCCTCCTCA |
| **12** | SPB-11335 | CATATTCAACCAGAATGATA |
|  | SPB-12067 | AATCGTTCTCCATTTGATTTTGC |
| **13** | SPB-11876 | CGAGGTAAAGTACCACGTACTCA |
|  | SPB-12595 | GTTGGATTTCTAACTTTATTRGARCG |
| **14** | SPB-12261 | TACCTCATAAGAAATAGTTTGAGC |
|  | SPB-13000 | TTACCTTAGGGATAACAGCGTAA |
| **15** | SPB-12888 | CCGGTCTGAACTCAGATCATGTA |
|  | SPB-13889 | ATTTATTGTACCTTTTGTATCAG |
| **16** | SPB-13342 | CCTTTGCACAGTCAAAATACTGC |
|  | SPB-14220 | TTATGCACACATCGCCCGTC |
| **17** | SPB-14197 | GTAAAYCTACTTTGTTACGACTT |
|  | SPB-14745 | GTGCCAGCAAYCGCGGTTATAC |
| **18** | SPB-14610 | ATAATAGGGTATCTAATCCTAGT |
|  | SPB-200 | ACCTTTATAAATGGGGTATGAACC |
| **19** | SPB-586 | CCATTCCATTTYTGATTTCC |
|  | SPB-1738 | TTTATTCGTGGAAATGCTATGTC |
| **20** | SPB-1709 | AATTGGTGGTTTTGGAAATTG |
|  | SPB-2776 | GGTAATCAGAGTATCGACG |
| **21** | SPB-2756 | ACATTTTTTCCTCAACATTT |
|  | SPB-3389 | TATTCATATCTTCAATATCATTGATG |
| **22** | SPC-10621 | CTCATACTGATGAAATTTTGGTTC |
|  | SPC-11526 | TTCTACTGGTCGTGCTCCAATTCA |
