# Supplementary material for: Comparative Mitogenomics of the Assassin Bug Genus Peirates (Hemiptera: Reduviidae: Peiratinae) Reveal Conserved Mitochondrial Genome Organization of P. atromaculatus, P. fulvescens and P. turpis
Source: PLoS One. 2015 Feb 17;10(2):e0117862. doi: 10.1371/journal.pone.0117862 (PMC4331094; doi:10.1371/journal.pone.0117862)
Supplement: S4 Table — (DOCX) [file pone.0117862.s009.docx]

**Table S4 The best partitioning scheme selected by PartitionFinder for the BI and ML analyses**

| **Phylogenetic methods** | **Subset Partitions** | **Best Model** |
| --- | --- | --- |
| BI: 5 partitions | P1: (12srRNA, 16srRNA, tRNA-Arg, tRNA-His, tRNA-Ile, tRNA-Phe, tRNA-Tyr) | GTR+G |
|  | P2: (ATP6, COI, COII, COIII, CytB) | GTR+I |
|  | P3: (ATP8, ND2, ND3, ND6, tRNA-Ala, tRNA-Asn, tRNA-Asp, tRNA-Cys, tRNA-Glu) | HKY+G |
|  | P4: (ND1, ND4, ND4L, ND5) | HKY+I |
|  | P5: (tRNA-Gln, tRNA-Gly, tRNA-Leu1, tRNA-Leu2, tRNA-Lys, tRNA-Met, tRNA-Pro, tRNA-Ser1, tRNA-Ser2, tRNA-Thr, tRNA-Trp, tRNA-Val) | HKY+I |
| ML: 4 partition | P1: (12srRNA, 16srRNA, tRNA-Arg, tRNA-Cys, tRNA-Gln, tRNA-Gly, tRNA-His, tRNA-Ile, tRNA-Leu1, tRNA-Leu2, tRNA-Lys, tRNA-Met, tRNA-Phe, tRNA-Pro, tRNA-Ser1, tRNA-Ser2, tRNA-Thr, tRNA-Trp, tRNA-Tyr, tRNA-Val) | GTR+G |
|  | P2: (ATP6, COI, COII, COIII, CytB) | GTR+G |
|  | P3: (ATP8, ND2, ND3, ND6, tRNA-Ala, tRNA-Asn, tRNA-Asp, tRNA-Glu) | GTR+G |
|  | P4: (ND1, ND4, ND4L, ND5) | GTR+G |
