# Supplementary material for: Comparative Mitogenomics of the Assassin Bug Genus Peirates (Hemiptera: Reduviidae: Peiratinae) Reveal Conserved Mitochondrial Genome Organization of P. atromaculatus, P. fulvescens and P. turpis
Source: PLoS One. 2015 Feb 17;10(2):e0117862. doi: 10.1371/journal.pone.0117862 (PMC4331094; doi:10.1371/journal.pone.0117862)
Supplement: S5 Table — (DOCX) [file pone.0117862.s010.docx]

**Table S5 Start and stop codons of protein-coding genes in *Peirates* mitochondrial genomes**

| **Gene** | **Start codon** | | | | | **Stop codon** | | | | |
| --- | --- | --- | --- | --- | --- | --- | --- | --- | --- | --- |
|  | **PF** | **PAY** | **PT** | **PA** | **PL** | **PF** | **PAY** | **PT** | **PA** | **PL** |
| *ND2* | ATG | ATG | ATG | ATG | ATG | TAA | TAA | TAA | TAA | TAA |
| *COI* | ATG | ATG | ATG | ATG | ATG | TAA | TAA | TAA | TAA | TAA |
| *COII* | ATC | ATC | ATC | ATC | ATC | T- | T- | T- | T- | T- |
| *ATP8* | ATA | ATA | ATA | **ATT** | **ATT** | TAA | TAA | TAA | TAA | TAA |
| *ATP6* | ATG | ATG | ATG | ATG | ATG | TAG | TAG | TAG | **TAA** | TAG |
| *COIII* | ATG | ATG | ATG | ATG | ATG | T- | T- | T- | T- | T- |
| *ND3* | ATT | ATT | ATT | ATT | ATT | T- | T- | T- | **TAA** | T- |
| *ND5* | ATT | ATT | ATT | ATT | ATT | T- | T- | T- | **TAA** | T- |
| *ND4* | ATG | ATG | ATG | ATG | ATG | TAA | TAA | TAA | TAA | TAA |
| *ND4L* | ATG | ATG | ATG | ATG | ATG | TAA | TAA | TAA | TAA | TAA |
| *ND6* | ATT | ATT | ATT | **ATA** | **ATC** | TAA | TAA | TAA | TAA | TAA |
| *CytB* | ATG | ATG | ATG | ATG | ATG | TAA | TAA | TAA | TAA | TAA |
| *ND1* | ATA | ATA | ATA | **ATG** | ATA | TAA | TAA | TAA | **TAG** | TAA |
