# Supplementary material for: Comparative Mitogenomics of the Assassin Bug Genus Peirates (Hemiptera: Reduviidae: Peiratinae) Reveal Conserved Mitochondrial Genome Organization of P. atromaculatus, P. fulvescens and P. turpis
Source: PLoS One. 2015 Feb 17;10(2):e0117862. doi: 10.1371/journal.pone.0117862 (PMC4331094; doi:10.1371/journal.pone.0117862)
Supplement: S6 Table — (DOCX) [file pone.0117862.s011.docx]

**Table S6 Genetic distances (%) calculation based on barcoding region, single PCG and concatenated 13 PCGs**

|  | **Within three assassin bugs*** | | |  | **Between three assassin bugs and other species** | | |
| --- | --- | --- | --- | --- | --- | --- | --- |
| **Gene** | **Mean** | **Min** | **Max** |  | **Mean** | **Min** | **Max** |
| Barcoding region | 3.1 | 1.5 | 4.1 |  | 13.8 | 11.9 | 16.1 |
| ATP6 | 3.6 | 1.6 | 4.9 |  | 14.6 | 13.5 | 16.2 |
| ATP8 | 4.5 | 2.0 | 6.1 |  | 27.1 | 18.4 | 38.1 |
| COI | 4.1 | 1.3 | 5.6 |  | 15.4 | 12.7 | 18.4 |
| COII | 2.6 | 0.4 | 3.8 |  | 15.7 | 13.5 | 18.2 |
| COIII | 3.6 | 1.4 | 4.9 |  | 14.4 | 12.5 | 15.8 |
| CytB | 4.0 | 2.0 | 5.1 |  | 16.1 | 14.0 | 18.5 |
| ND1 | 4.7 | 2.5 | 6.5 |  | 13.5 | 12.7 | 14.4 |
| ND2 | 3.2 | 1.1 | 4.5 |  | 18.8 | 15.4 | 22.6 |
| ND3 | 4.2 | 0.6 | 6.3 |  | 18.8 | 12.9 | 23.4 |
| ND4 | 3.8 | 2.1 | 4.7 |  | 12.3 | 10.8 | 13.9 |
| ND4L | 4.1 | 1.4 | 5.9 |  | 16.6 | 13.9 | 19.7 |
| ND5 | 4.0 | 1.9 | 5.3 |  | 13.9 | 11.6 | 15.9 |
| ND6 | 3.7 | 1.8 | 4.8 |  | 14.9 | 10.8 | 18.7 |
| Concatenated 13 PCGs | 3.8 | 1.6 | 5.0 |  | 15.0 | 13.0 | 17.0 |

* PF, PAY and PT.
