# Supplementary material for: Comparative Mitogenomics of the Assassin Bug Genus Peirates (Hemiptera: Reduviidae: Peiratinae) Reveal Conserved Mitochondrial Genome Organization of P. atromaculatus, P. fulvescens and P. turpis
Source: PLoS One. 2015 Feb 17;10(2):e0117862. doi: 10.1371/journal.pone.0117862 (PMC4331094; doi:10.1371/journal.pone.0117862)
Supplement: S7 Table — (DOCX) [file pone.0117862.s012.docx]

**Table S7 Statistics on gene overlaps in *Peirates* mitochondrial genomes**

| **Species** | ***trnI*-**  ***trnQ*** | ***ND2-***  ***trnW*** | ***trnW-***  ***trnC*** | ***COI-***  ***trnL2*** | ***trnK-***  ***trnD*** | ***ATP8-***  ***ATP6*** | ***ATP6-***  ***COIII*** | ***trnR-***  ***trnN*** | ***trnE-***  ***trnF*** | ***trnF-***  ***ND5*** | ***trnH-***  ***ND4*** | ***ND4-***  ***ND4L*** | ***ND6-***  ***CytB*** | ***CytB-***  ***trnS2*** | ***ND1-***  ***trnL1*** |
| --- | --- | --- | --- | --- | --- | --- | --- | --- | --- | --- | --- | --- | --- | --- | --- |
| PF | **3** | **-** | **8** | **5** | **1** | **7** | **44** | **2** | **-** | **-** | **1** | **7** | **1** | **1** | **6** |
| PAY | **3** | **-** | **8** | **5** | **1** | **7** | **44** | **2** | **-** | **-** | **1** | **7** | **1** | **1** | **6** |
| PT | **3** | **-** | **8** | **5** | **1** | **7** | **44** | **2** | **-** | **-** | **1** | **7** | **1** | **1** | **6** |
| PL | 3 | - | **8** | **5** | - | **7** | 44 | - | - | - | - | **7** | **1** | - | 6 |
| PA | 22 | 2 | **8** | **5** | 1 | **7** | 17 | - | 2 | 1 | 1 | **7** | **1** | - | - |
