# Supplementary material for: Comparative Mitogenomics of the Assassin Bug Genus Peirates (Hemiptera: Reduviidae: Peiratinae) Reveal Conserved Mitochondrial Genome Organization of P. atromaculatus, P. fulvescens and P. turpis
Source: PLoS One. 2015 Feb 17;10(2):e0117862. doi: 10.1371/journal.pone.0117862 (PMC4331094; doi:10.1371/journal.pone.0117862)
Supplement: S8 Table — (DOCX) [file pone.0117862.s013.docx]

**Table S8 Statistics on non-coding regions in *Peirates* mitochondrial genomes**

| **Species** | ***trnQ*-**  ***trnM*** | ***ND2-***  ***trnW*** | ***trnC-***  ***trnY*** | ***trnY*-**  ***COI*** | ***ND3*-**  ***trnA*** | ***trnA-***  ***trnR*** | ***trnR*-**  ***trnN*** | ***trnE –***  ***trnF*** | ***ND4L-***  ***trnT*** | ***trnP-***  ***ND6*** | ***CytB-***  ***trnS2*** | ***trnS2 –***  ***ND1*** | ***ND1-***  ***trnL1*** |
| --- | --- | --- | --- | --- | --- | --- | --- | --- | --- | --- | --- | --- | --- |
| PF | **22** | **54** | **8** | **1** | **-** | **3** | **-** | **2** | **3** | **2** | **-** | **22** | **-** |
| PAY | **22** | **54** | **8** | **1** | **-** | **3** | **-** | **2** | **3** | **2** | **-** | **22** | **-** |
| PT | **22** | **54** | **8** | **1** | **-** | **3** | **-** | **2** | **3** | **2** | **-** | **22** | **-** |
| PL | **22** | 36 | 6 | **1** | - | **3** | - | 3 | 2 | 2 | 4 | **22** | - |
| PA | **22** | - | 3 | **1** | 1 | **3** | 4 | - | 3 | 5 | 9 | **22** | 1 |
