# Supplementary material for: Comparative Mitogenomics of the Assassin Bug Genus Peirates (Hemiptera: Reduviidae: Peiratinae) Reveal Conserved Mitochondrial Genome Organization of P. atromaculatus, P. fulvescens and P. turpis
Source: PLoS One. 2015 Feb 17;10(2):e0117862. doi: 10.1371/journal.pone.0117862 (PMC4331094; doi:10.1371/journal.pone.0117862)
Supplement: S9 Table — (DOCX) [file pone.0117862.s014.docx]

**Table S9 Sequence identities of structural elements in control region**

|  | **Leading sequence** | **AT-rich sequence** | **The remainder of control region** |
| --- | --- | --- | --- |
| Three assassin bugs* | 98.5% | 96.46% | 91.13% |
| All species | 70.37% | 15.47% | 57.58% |

* PF, PAY and PT.
